# Perception and control of low cable operation forces in voluntary closing body-powered upper-limb prostheses

**DOI:** 10.1371/journal.pone.0225263

**Published:** 2019-11-22

**Authors:** Mona Hichert, David A. Abbink, Alistair N. Vardy, Corry K. van der Sluis, Wim G. M. Janssen, Michael A. H. Brouwers, Dick H. Plettenburg

**Affiliations:** 1 Department of Biomechanical Engineering, Delft University of Technology, Delft, The Netherlands; 2 Department of Cognitive Robotics, Delft University of Technology, Delft, The Netherlands; 3 University of Groningen, University Medical Center Groningen, Department of Rehabilitation Medicine, Groningen, The Netherlands; 4 Rijndam Rehabilitation Centre Rotterdam, Department of Rehabilitation Medicine Erasmus MC, Rotterdam, The Netherlands; 5 Rehabilitation Institute De Hoogstraat, Utrecht, The Netherlands; Washington University in Saint Louis School of Medicine, UNITED STATES

## Abstract

Operating a body-powered prosthesis can be painful and tiring due to high cable operation forces, illustrating that low cable operation forces are a desirable design property for body-powered prostheses. However, lower operation forces might negatively affect controllability and force perception, which is plausible but not known. This study aims to quantify the accuracy of cable force perception and control for body-powered prostheses in a low cable operation force range by utilizing isometric and dynamic force reproduction experiments. Twenty-five subjects with trans-radial absence conducted two force reproduction tasks; first an isometric task of reproducing 10, 15, 20, 25, 30 or 40 N and second a force reproduction task of 10 and 20 N, for cable excursions of 10, 20, 40, 60 and 80 mm. Task performance was quantified by the force reproduction error and the variability in the generated force. The results of the isometric experiment demonstrated that increasing force levels enlarge the force variability, but do not influence the force reproduction error for the tested force range. The second experiment showed that increased cable excursions resulted in a decreased force reproduction error, for both tested force levels, whereas the force variability remained unchanged. In conclusion, the design recommendations for voluntary closing body-powered prostheses suggested by this study are to minimize cable operation forces: this does not affect force reproduction error but does reduce force variability. Furthermore, increased cable excursions facilitate users with additional information to meet a target force more accurately.

## Introduction

Body-powered prostheses are operated by body movements of the user, typically transferred by a shoulder harness to operate the prosthetic end-effector that generates the grasping forces. In a voluntary closing prosthesis the grasping forces are generated by the operators’ shoulder movements; in a voluntary opening body-powered prosthesis the grasping forces are generated by a spring that the operator needs to pull on to open the prosthesis. Voluntary closing prostheses are regarded to be better suited for handling delicate or breakable objects than voluntary opening prostheses, since the pinch force is controlled directly by the user instead of the spring.

Body-powered prostheses offer bi-directional force interaction through the mechanical linkage, meaning that end-effector positions and grasping forces generate proprioceptive feedback in the shoulder and arm muscles. This feedback is also called Extended Physiological Proprioception (EPP), as coined by Simpson [1]. Literature has shown that proprioceptive feedback allows higher bandwidths of motor control tasks, compared to visual and tactile feedback [2]. In contrast, myo-electric prostheses require their users to rely mostly on visual feedback to estimate end-effector position: although auditory and tactile feedback from motor vibrations serve as a rough estimate of grasping forces, this does not offer the high bandwidth proprioceptive information of body powered prostheses. The enhanced feedback due to EPP of body-powered prostheses results in substantially improved control of pinch forces during object manipulation.

One of the limitations of both voluntary closing and opening body-powered prostheses is that currently available hands and hooks require high operation forces [3,4] and cannot supply sufficient grip strength [5]. High operation forces contribute to the relatively high rejection rates of body-powered prostheses [6]. Requirements for forces range from a 20 N pinch force to be sufficient for most daily activities [7,8] to 34 N pinch force [9] for pulling on a sock. Such force requirements require high forces from the shoulder movements in today’s commercially available voluntary closing devices: these require between 33 and 131 N of cable force to generate 15 N of pinch force [3]. Also voluntary opening devices configured to pinch similar force levels require between 50 and 120 N to open [4]. Such high force levels unfortunately may lead to fatigue or painful use [5]. Furthermore, some potential prosthesis users are not even capable to generate these high cable forces [10].

Fatigue and pain are not the only downsides of high cable operation forces: in voluntary closing prostheses high cable forces can also deteriorate pinch force control accuracy [11]. Locking mechanisms like the TRS SURE-LOK allow the user of a voluntary closing prosthesis to relax the cable force while holding objects for longer periods of time [12], but this does not allow easy adjustments in force during manipulation.

In short, it is desirable to decrease the cable operating forces, but by how much? An upper bound on the cable operating forces is available from literature: the fatigue-free cable operating force range is below 38 N for the average female user and below 66 N for the average male user [10]. But what should be the lower bound? What is the impact of lowering cable operating forces on force accuracy and perception, and how does this relate to design choices for cable excursions (which currently range for voluntary closing prostheses from 37 – 60 mm and for voluntary opening prostheses from 22 – 56 mm). Increased cable operation excursions may play a role in transmitting lower cable forces to higher pinch forces. Neither the effect of low cable forces nor that of cable excursions on the perception and control of body-powered prostheses have been investigated before.

This study aims to establish design guidelines for a ‘preferred window’ of cable forces and excursions that allows pain-free, fatigue-free operation of body powered prostheses that provide optimal perception and control. In order to make such guidelines generalizable to the control of different types of end-effector prosthetics, we aim to establish this ‘preferred window’ for the cable operation forces. Our approach is to use static force reproduction experiments to investigate systematic force reproduction errors, i.e., the difference of target force, perceived in a first trial, and reproduced force, generated in the following trial. The force reproduction error can be interpreted as the difference between the intended and the created cable force (that relates directly to the pinch force through end-effector dynamics) on an object and is therefore a measure for successful object manipulation with a voluntary closing prosthesis.

This study builds on a preliminary force reproduction experiment, which was isometric (i.e. no cable excursions) and in which only few prosthesis users could participate [13]. In that work, for healthy controls (n = 13) the force reproduction errors were found to be lowest between 20-30 N, and for prosthesis users (n = 7) between 10-20 N. Also, increasing force levels increased force variability, in correspondence to the well-established relationship between force variability due to motor noise and increasing force levels [14,15]. With respect to that preliminary study, the contribution of the present study is including various cable excursion design options when quantifying the force reproduction errors, for a representative group of prosthesis users.

The specific goal of this study was to quantify the accuracy of cable force perception and control when using a body-powered prosthesis with a low cable operation force range, by means of (isometric and dynamic) force reproduction experiments. We empirically test three hypotheses. First, we hypothesize that cable forces between 10-20 N will result in the smallest force reproduction error, based on preliminary experiments [13]. Second, we hypothesize that at cable operation forces of 10 and 20 N, increasing cable excursions will decrease force reproduction error. This hypothesis is based on motor control literature which suggests that position information in addition to the perceived and controlled forces may decrease the force reproduction error [16]. Third, we hypothesize that the force variability will increase with increasing target force levels.

## Methods

### Approach

Force reproduction experiments either request subjects to reproduce a force generated on the participant [17,18], or reproduce a self-generated force [19,20]. We choose the second, to let the subject first reproduce a target force which is illustrated visually on a screen (visual block), and consequentially receiving proprioceptive feedback of his body movements and tactile feedback of the exerted forces on the skin by prosthetic parts (harness and socket). Based on the perceived forces the subject reproduces the same force again without visual information (blind block). It is mainly the proprioceptive feedback perceived during the visual block, which enables the user to reproduce the same force during the blind block [16]. This simulates prosthesis use: the user estimates a force required to manipulate an object (experimental: target force) and based on his experience of former perceived forces (experimental: visual blocks), he applies the required force (experimental: blind block).

The experimental setup should be unaffected by (mechanical) properties of available prehensors and therefore either 1) a threaded rod or 2) springs of different stiffness were mounted on the end of the control cable instead of a voluntary closing prehensor. The threaded rod setting, as used in the ‘no cable excursion trials’, simulates holding a rigid object with a voluntary closing prehensor at a constant cable excursion. The “variable-spring-stiffness” setting, as used in the ‘cable excursion trials’, simulates the approach of a desired pinch force to hold an object with a voluntary closing prehensor.

Cable forces of interest were based on the examined cable force levels on TRS hook data of Smit and Plettenburg’s study [3], since the TRS hook requires the lowest cable force of all tested devices. At 10 N the TRS hook starts building up a pinch force. At 40 N the TRS hook pinches approximately 20 N. A pinch force of 20 N is reported to be sufficient to complete most daily activities with an upper-limb prosthesis [7,8]. Additionally, the critical force, which is the force humans can exert without fatigue effects during continuous isometric contractions, should be considered as upper force boundary for prosthesis use. Monod determined the critical force at 15 and 20% of the maximum voluntary contraction [21]. Considering maximum cable operation forces reported by Taylor (arm flexion: 280 (24) N; shrug: 270 (106) N; arm extension: 251 (29) N) [22] and Hichert et al. (combination of shoulder protraction, humeral abduction and flexion: 267 (123) N) [23] as maximum voluntary contraction, the target forces should not exceed 40 N (251 N x 15%) to enable participants to complete all trials. Based on this, we decided to examine six force levels (10, 15, 20, 25, 30 and 40 N) for the threaded rod setting.

In contrast to the reported magnitude of maximum cable excursion of 58 (1.7) mm for arm extension [22] by Taylor, we measured maximum cable excursions of 160 to 260 mm in preliminary experiments. These experiments also showed that up to 50% of the maximum cable excursion the subjects’ operation force levels were unchanged. The cable excursion should therefore not exceed 80 mm (160 mm x 50%). Based on this, we decided to examine five cable excursions (10, 20, 40, 60 and 80 mm) for the “variable-spring-stiffness” setting. The five excursions were tested at two force levels, 10 and 20 N, at which the crossover point from overestimation to underestimation of target forces for prosthesis users were found in preliminary experiments [13]. This results in ten force-excursion conditions.

### Participants

Twenty-four adults (12 females, age: 49 (13) years, height: 175 (8) cm, weight: 75 (14) kg) with congenital and acquired unilateral trans-radial defects participated. All participants were free of neurological, muscle, joint or motor control problems concerning the upper extremity or the torso (exclusion criteria). A total of 16 participants had a left deficiency, 15 had a congenital defect, 13 had experience with body-powered prostheses and five are current body-powered prosthesis users.

This study was approved by the medical ethical committee of University Medical Centre Groningen (UMCG) (NL41112.042.12). The participants were recruited from University Medical Center Groningen, Erasmus Medical Center, Rotterdam, and the rehabilitation institute De Hoogstraat, Utrecht.

### Materials

**Fig 1:**
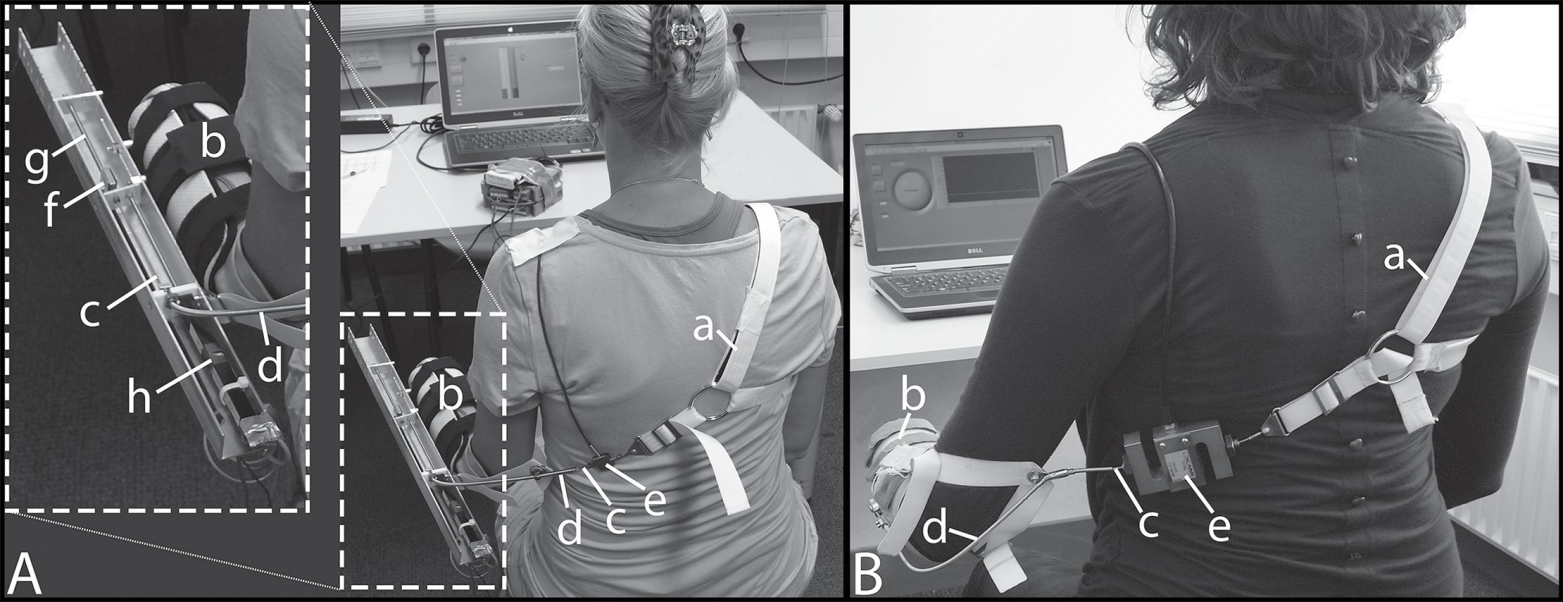
Measurement set-up. The measurement set-up for the force reproduction task (A) and maximum force measurements (B) consisted of a “figure-of –nine” harness (a) and thermoplastic shell (b) which are connected through a Bowden cable (c) running through a cable housing (d). In the maximum force measurements setup (B) the cable excursion is disabled. Here the cable (c) is interrupted by a force sensor at the subject’s back (e). In the force reproduction task setup the cable is interrupted by two force sensors (e & f), which measure the cable forces before (F_back_) and after (F_arm_) the cable housing respectively. In this figure a threaded rod (g) is illustrated leading to disabled cable excursions. The threaded rod is interchangeable with springs of different stiffness, which resulted in different cable force-excursion characteristics. A displacement sensor is recording cable excursions (h).

A custom-made prosthesis simulator (Fig 1A) was connected to the participant’s prosthesis by a thermoplastic shell. For two participants, who did not own a prosthesis, the prosthesis simulator was placed on a temporary WILMER Open Fitting socket [7]. For two other participants the prosthesis simulator was attached to the remnant arm since its length was sufficient for a firm connection. The prosthesis simulator consisted of an adjustable “figure-of-nine” harness linked to a standard 1/16’’ (.159 cm) diameter stainless steel cable (C100, Hosmer Dorrance Corporation, Chattanooga, USA). The end of the control cable, which was positioned in a U-profile, was attached to either 1) a threaded rod or 2) springs of different stiffness. The steel cable was interrupted by two force sensors (FLLSB200 222 N, FUTEK, Irvine, USA), one before and one after the stainless steel cable housing for C-100HD cable (CH-100HD, Hosmer Dorrance Corporation, Chattanooga, USA). To decrease friction in the cable a Teflon liner for heavy duty cable housing (CH100-HD, Hosmer Dorrance Corporation, Chattanooga, USA) was placed in the inside of the cable housing. A U-profile was fixated to the thermoplastic shell. Within the U-profile a displacement sensor (13FLP100 A, Sakae, Zhejiang, China) was placed. The two force sensors were amplified (CPJ, Scaime, Juvigny, France) and sampled together with the displacement sensor at 50 Hz (NI USB-6008, National Instruments, Austin, USA), and finally stored using a custom LabVIEW program (LabVIEW 2012, National Instruments, Austin, USA).

To investigate ten different force-excursion conditions, ten interchangeable springs with varying spring stiffness and pretensions were utilized as shown in Table 1. The spring’s characteristics (stiffness and pretension) were chosen to match the predefined force-excursion parameters.

**Table 1:** Stiffness and pretension of the utilized springs in each condition.

Condition	Spring stiffness [N/mm]	Spring pretension [N]
10 N – 10 mm	0.44 (0.06)	5.5 (0.6)
10 N – 20 mm	0.19 (0.04)	6.3 (0.8)
10 N – 40 mm	0.20 (0.01)	2.0 (0.3)
10 N – 60 mm	0.08 (0.00)	5.6 (0.1)
10 N – 80 mm	0.08 (0.00)	4.0 (0.1)
20 N – 10 mm	1.50 (0.18)	5.3 (1.6)
20 N – 20 mm	0.57 (0.01)	8.9 (0.2)
20 N – 40 mm	0.26 (0.01)	10.0 (0.1)
20 N – 60 mm	0.22 (0.00)	7.2 (0.1)
20 N – 80 mm	0.21 (0.04)	5.2 (1.1)

#### Maximum force measurements

Another similar custom-made prosthesis simulator (Fig 1B) was utilized to measure the participants’ pre and post experimental maximum forces. Cable excursions were disabled in this setup. The Bowden cable was interrupted by a force sensor (S-Beam load cell ZFA 100kg, Scaime, Juvigny, France). The measured forces were amplified (CPJ, Scaime, Juvigny, France), sampled at 1 kHz (NI USB-6008, National Instruments, Austin, USA), and finally stored using a custom LabVIEW program (LabVIEW 2012, National Instruments, Austin, USA).

#### Questionnaires

To analyze the given task and the used system with its force –excursion combinations and the differences between the different conditions, subjective data of perceived workload were gathered via the Nasa Task Load Index (NASA-TLX) questionnaire (Desktop Version 2.1.2, developed by David Sharek, NASA Ames Research Center, Moffett Field, USA). A Dutch translation of the questionnaire was provided. The questionnaire assesses the total workload divided into six subscales: Mental Demand, Physical Demand, Temporal Demand, Performance, Effort, and Frustration.

**Fig 2:**
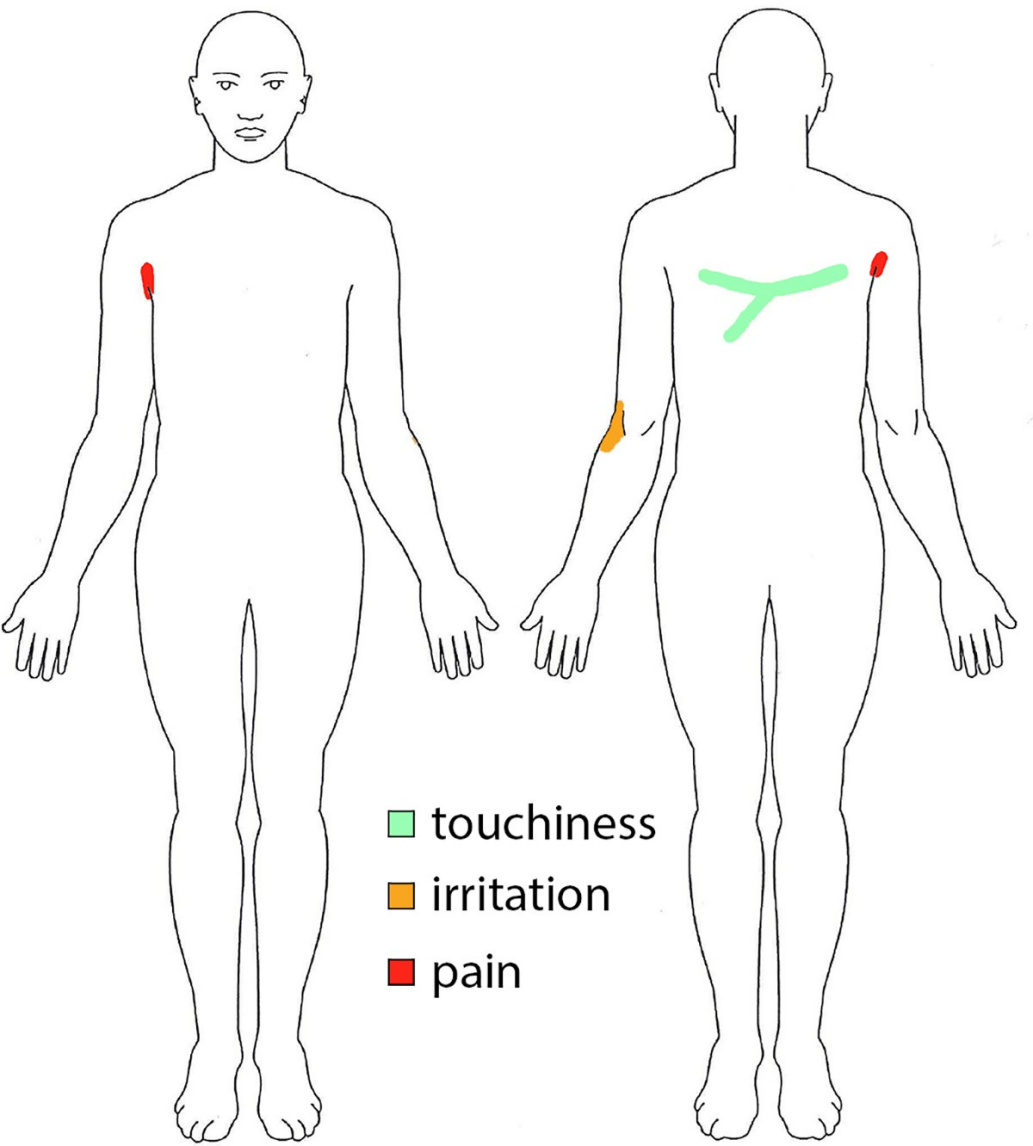
Body-map. The Body-Map was coloured by one subject indicating pain (red) at the back of the left elbow, irritation (orange) in the right arm pit and touchiness (green) on a stripe of his back.

Furthermore, subjects were requested to indicate regions of no, mild or severe discomfort on a map of the body (Body-Map) by coloring the respective body parts green (touchiness), orange (irritation), or red (pain) (Fig 2).

To monitor post experimental pain and fatigue effects, a few days after the experiment each participant was asked in an email whether he/she had experienced any post-experimental pain the day of the measurement or the following days, and if so in which part of the body.

### Procedure

**Fig 3:**
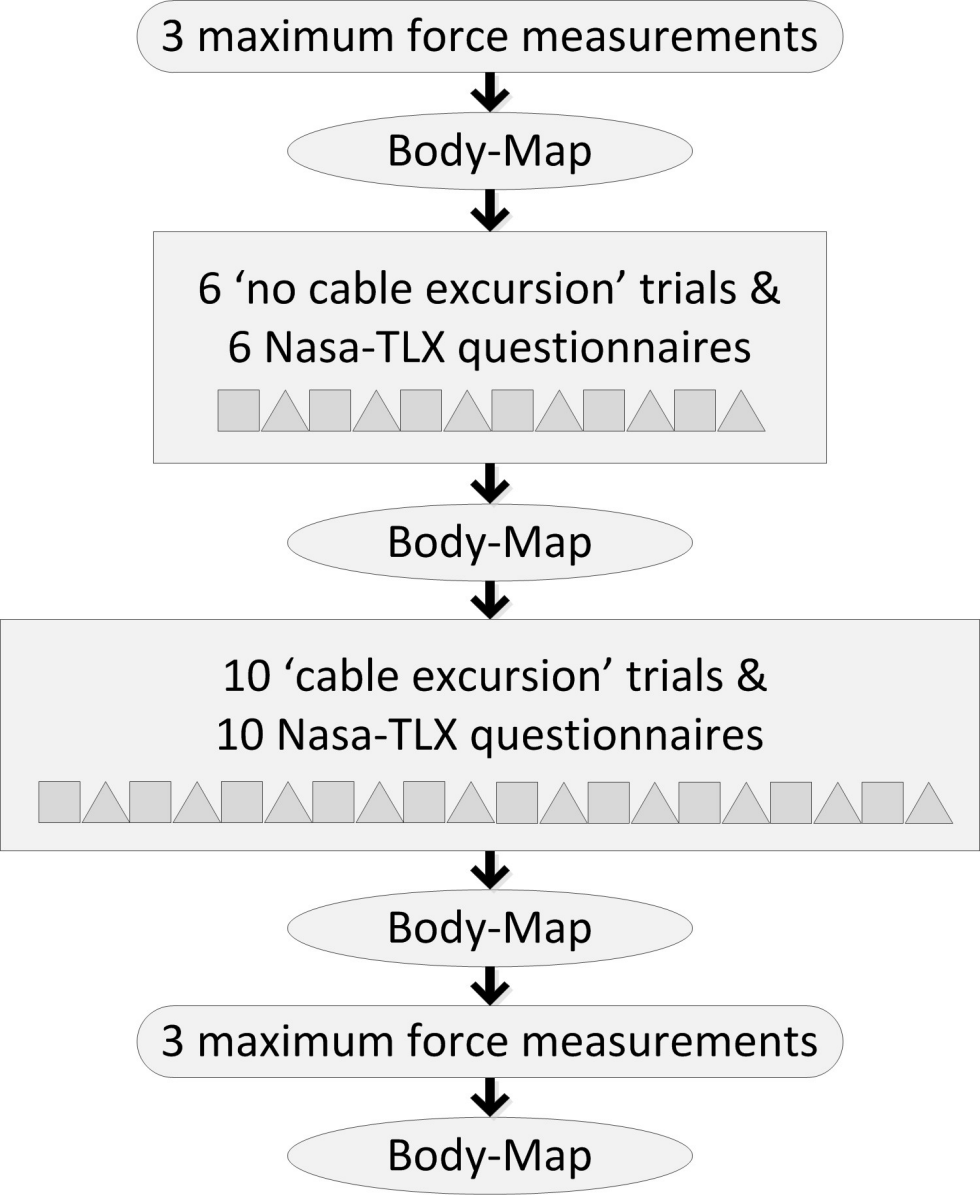
Experimental procedure. The flowchart of the experimental procedure illustrates the chronologic order of maximum force measurements (rounded rectangles), body-map questionnaires (circles), force reproduction experiments with and without cable excursion (rectangles) with alternating force reproduction trials (squares) and Nasa-TLX questionnaires (triangles).

The chronological experimental procedure is shown in Fig 3. First, subjects were requested to exert their maximum force on the cable utilizing the equipment shown in Fig 1B. Three measurements were taken with a duration of three seconds each. This procedure was repeated at the end of the experimental procedure to monitor physical fatigue caused by the experiment. Then the subjects conducted the force reproduction experiments equipped with the measurement setup shown in Fig 1A, consisting of two parts: six trials with cable excursion disabled, followed by ten trials with cable excursion. After completing each of these 16 trials the subject was requested to fill in a Nasa-TLX questionnaire. The individual relevance of each of the six subscales to the total workload was supplemented by a paired comparison of the six subscales, ascertained during the first and last questionnaire. The Body-Map questionnaire was provided four times: after the pre and post maximum force measurements as well as after the force reproduction experiments without and with cable excursion.

**Fig 4:**
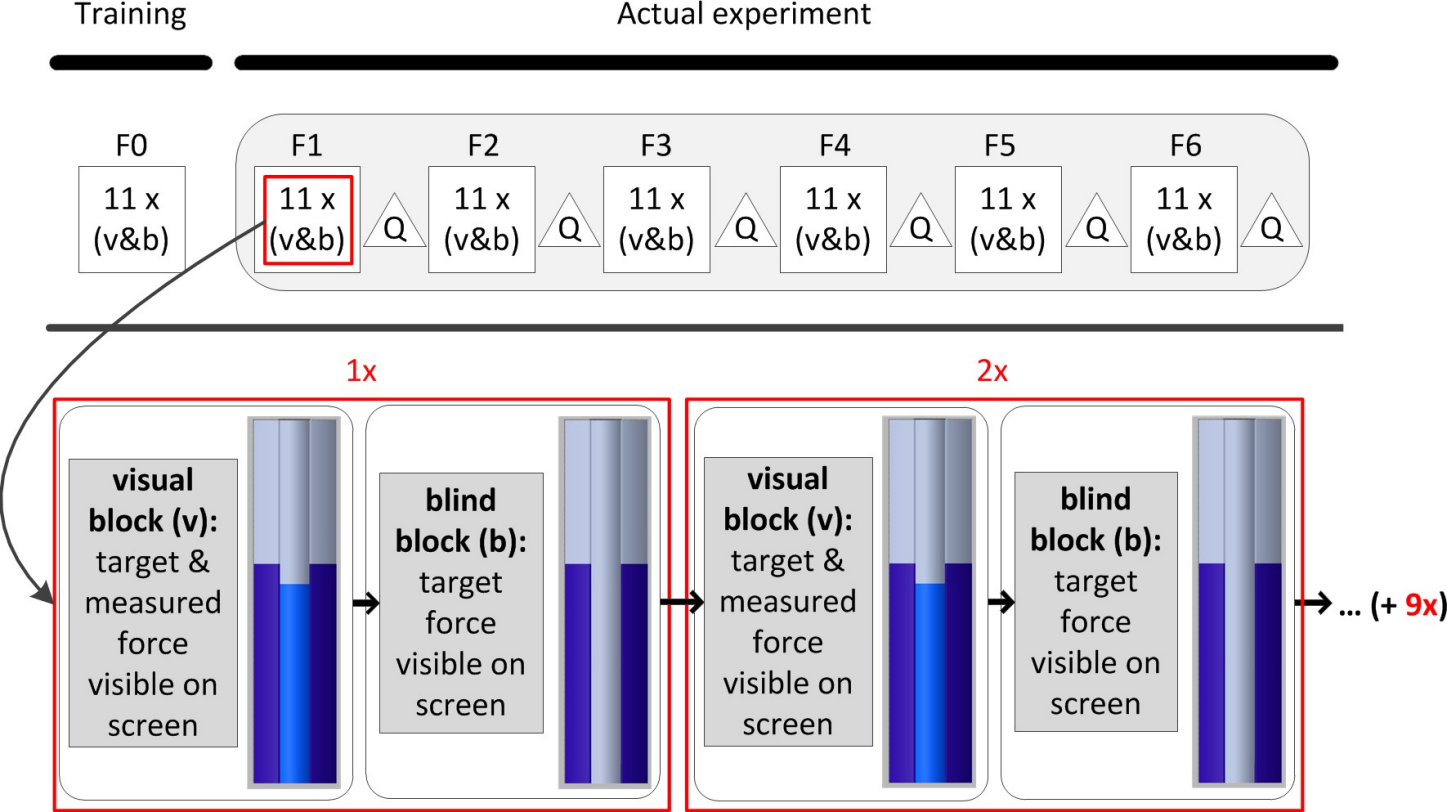
Experimental procedure of ‘no cable excursion’ trials. Flowchart illustrating the experimental procedure of the six ‘no cable excursion’ trials as shown in Fig 3. After practicing the force reproduction task at 22 N (F0), six force levels (10, 15, 20, 25, 30 and 40 N) were examined during 11 alternating visual and blind blocks. The force reproduction task at each force level (squares) was followed by a Nasa-TLX questionnaire (triangle). The order of force levels (F1 to F6) was counterbalanced over the subjects. The outer (purple) bars indicate the target force; the inner (blue) bar indicates the measured force.

For the force reproduction trials the measurement set-up of Fig 1A was fitted to the subject. During the ‘no cable excursion’ trials a threaded rod was placed in the U-profile disabling cable excursion. For the ‘cable excursion’ trials, the threaded rod was replaced by linear springs of different stiffnesses. Six force levels (10, 15, 20, 25, 30 and 40 N) for the ‘no cable excursion’ trials and ten force-excursion combinations (10, 20, 40, 60 and 80 mm each at 10 and 20 N) for the ‘cable excursion’ trials were examined resulting in 16 test conditions. Before each trial, the subject was allowed one training run at 22 N to familiarize himself with the task. Fig 4 shows the experimental procedure of the six ‘no cable excursion’ trials. The order of the six force levels (part 1 - ‘no cable excursion’ trials) and the ten force-excursion conditions (part 2 - ‘cable excursion’ trials) were counterbalanced over participants. One trial consisted of eleven alternating visual and blind alternating blocks, all at the same constant reference force level. So for example, the target force was 0 than 20 than 0 than 20-0-20 etc. One block lasted 5 seconds followed by a 2 second break, resulting in a duration of 152 seconds per trial. During a visual block the constant reference force and the produced force measured on the arm of the subject (F_arm_) was shown on the laptop screen, whereas during a blind block only the constant target force was displayed. In other words, during the visual blocks subjects reproduced the target force based on the visual information on the screen, whereas during the blind blocks subjects based the magnitude of the reproduced force on the perceived force during a visual block. Participants were instructed to produce the force as stable as possible. During the ‘cable excursion’ trials visual feedback to the subjects’ arm was disabled with a hairdressers cloth tightened to the walls, as the arm position would have given information about the cable excursion. Subjects had the opportunity to practice the given task for 120 seconds. For the ‘cable excursion’ trials subjects were given 60 seconds to become accustomed to the new condition. In the event that a subject experienced (concentration) difficulties in one block, another visual and blind block was added to the condition to complete the measurement.

### Data analysis

#### Metrics

Participants’ performance was assessed by the force reproduction error, which is the difference between target and reproduced force, and the force variability, which is the noise of the reproduced force. These metrics were determined from the cable forces measured at the back of the subject.

**Fig 5:**
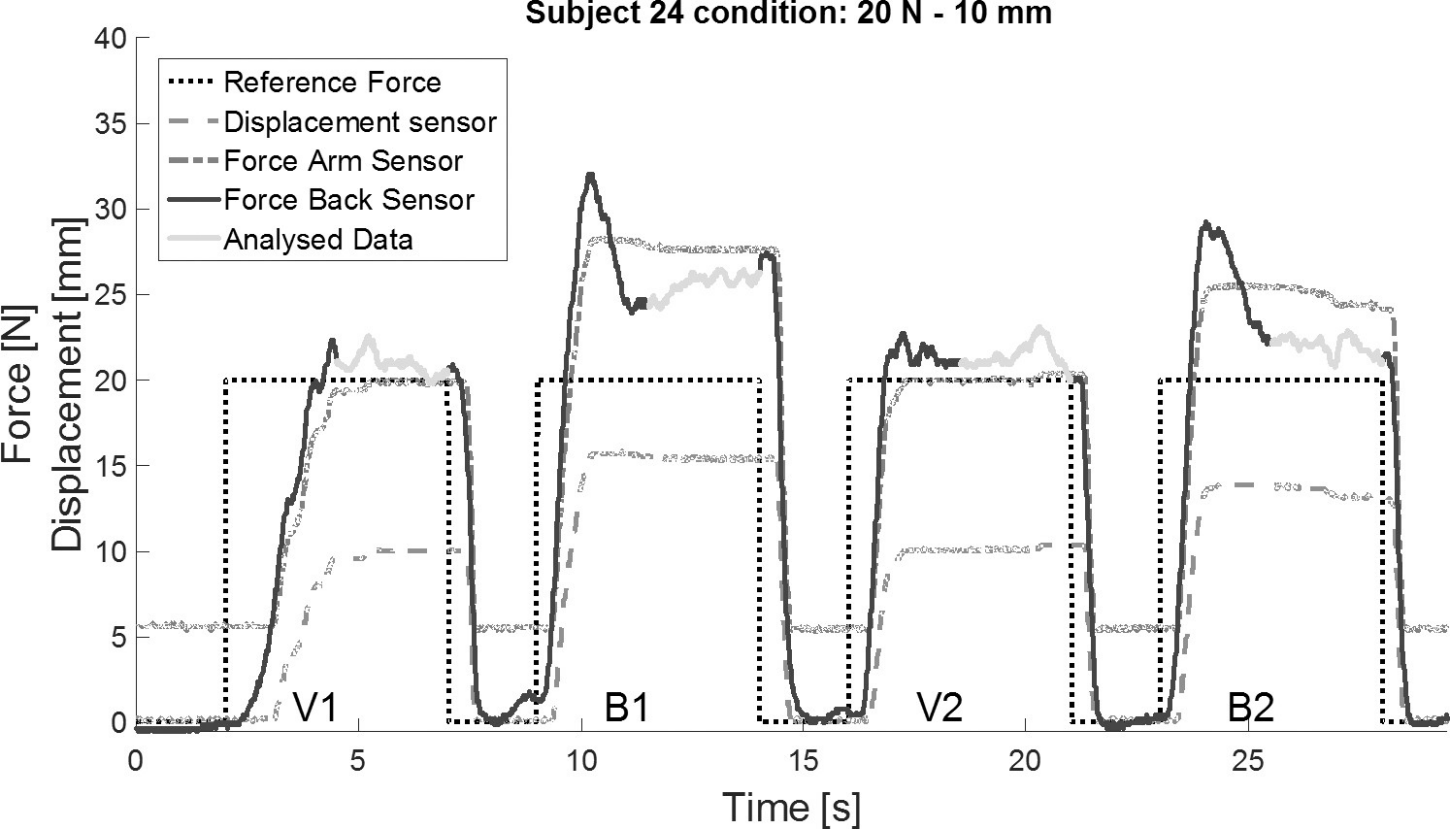
Raw data ‘cable excursion’ trials. The raw data of the first 30 seconds of a typical trial, condition 20 N – 10 mm, represents the target force of 20 N, the approximate 10 mm cable excursion measured by the displacement sensor and the two cable forces measured at the arm (F_arm_) and the back (F_back_) of the subject. Visual blocks (V1, V2) are alternating with blind blocks (B1, B2).

The last 2.5 seconds of measured force (Fig 5) were analyzed by calculating the mean and standard deviation. Because the perceived force during the visual block must be reproduced during the blind block, the force reproduction error (FRE) per block was calculated as the average force of a blind block minus the average force of the foregoing visual block (S1 Equation). The results per block were then averaged over all blocks of the trial to obtain the overall force reproduction error (per subject, per force level) (S2 Equation). The first visual and blind blocks of each trial were eliminated from data analysis.

The force variability (FV) results from the standard deviation of the blind blocks (S3 Equation) averaged over all analyzed blocks (S4 Equation).

The force reproduction error and force variability were determined for each condition (six force levels for ‘no cable excursion’ and ten force-excursion combinations for ‘cable excursion’ trials).

#### Maximum force measurements

The highest values of the three pre and three post maximum force measurements were determined. Only trials where the maximum force was attained within the predetermined 3 seconds were included (114 of 150 trials). In those cases where the measured cable force was still increasing at the 3 second mark, it was concluded that the maximum force had not yet been reached and the trial was excluded from the analyses. The maxima of the three pre and post measurements were taken to analyze for fatigue effects.

#### Statistics

For statistical analysis SPSS version 20 was used. Pre and post experiment maximal force levels were compared using a paired Student t-test. Repeated measures ANOVAs were used to determine the experimental effects (‘no cable excursion’ trials: target force; ‘cable excursion’ trials: target force × excursion) for force reproduction error and force variability. A significance level of α = 0.05 was maintained.

## Results

**Fig 6:**
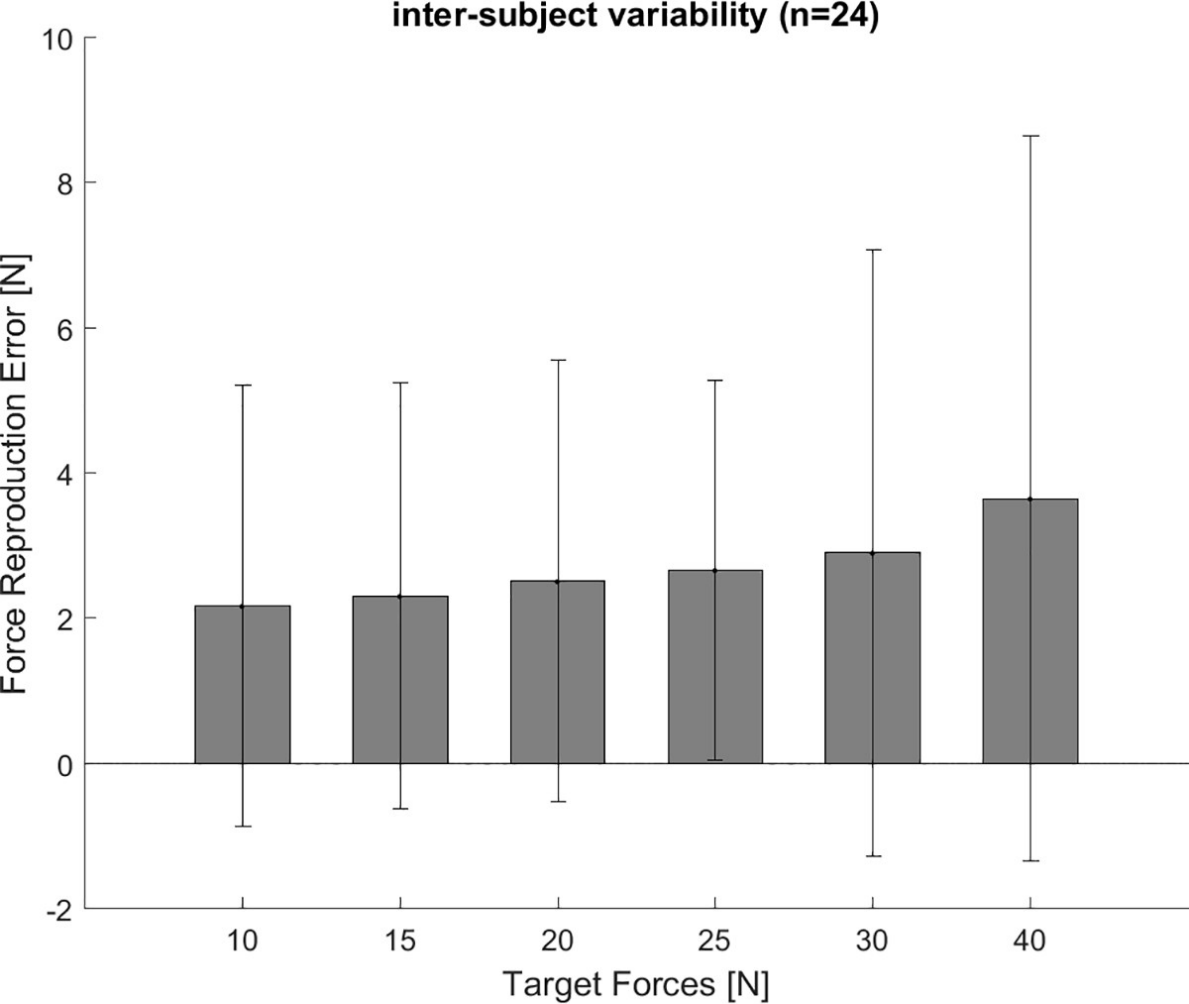
Force reproduction error ‘no cable excursion’ trials. The force reproduction error for the ‘no cable excursion’ trials shows no significant differences between the tested conditions of target forces between 10 and 40 N. The bars indicate the group’s average and the whiskers the standard deviation.

**Fig 7:**
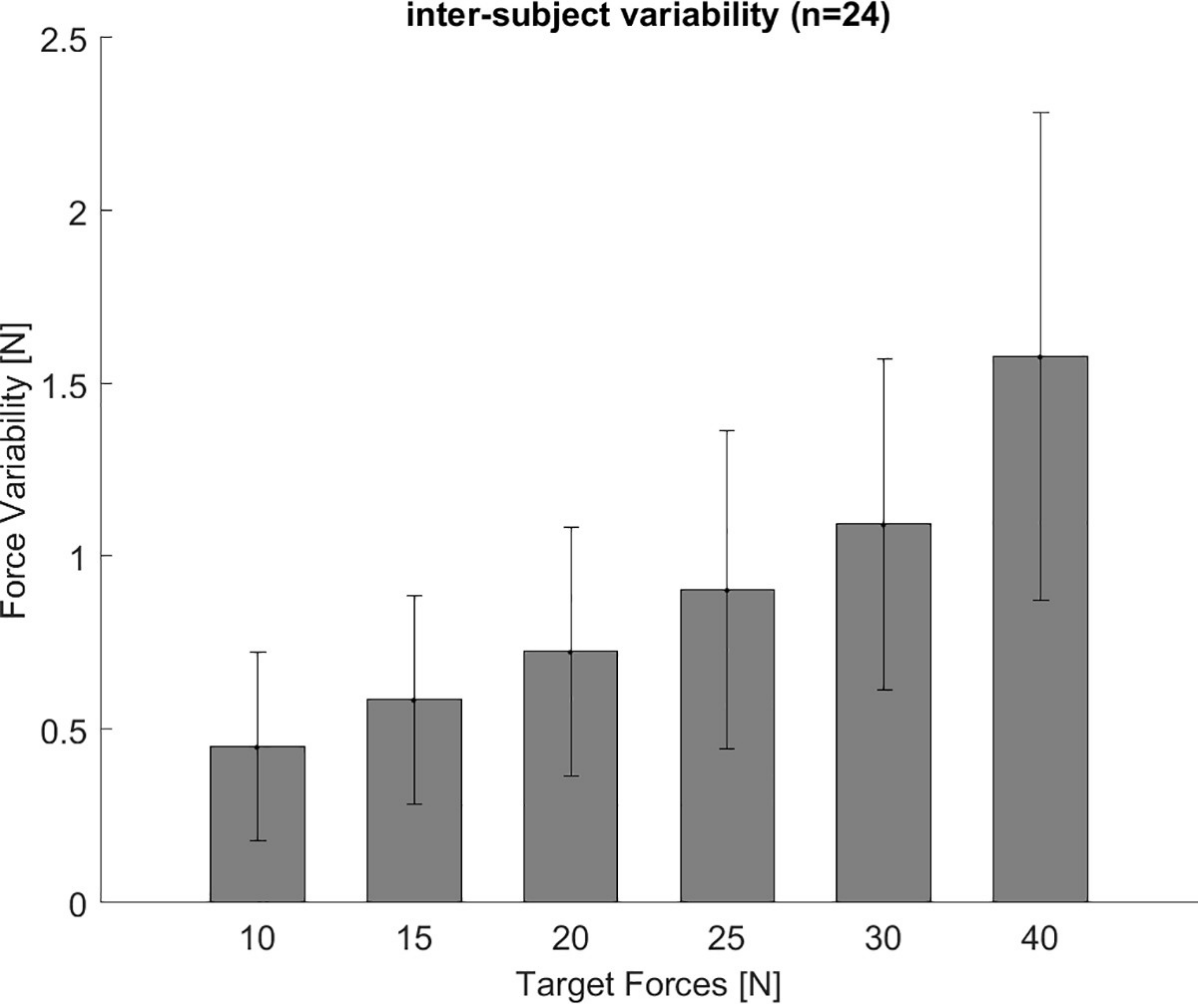
Force variability ‘no cable excursion’ trials. The force variability increases with increasing target force for the ‘no cable excursion’ trials. The bars indicate the group’s average and the whiskers the standard deviation.

The force reproduction error for ‘no cable excursion’ trials showed no difference for the measured target forces between 10 and 40 N (F(5,19) = 0.936, p = 0.48) (Fig 6). The target force was overestimated for all force levels, and consequentially we did not find a crossover point from over- to underestimation. With increasing target force the force variability was increasing (F(5,19) = 23.767, p<0.001) (Fig 7).

**Fig 8:**
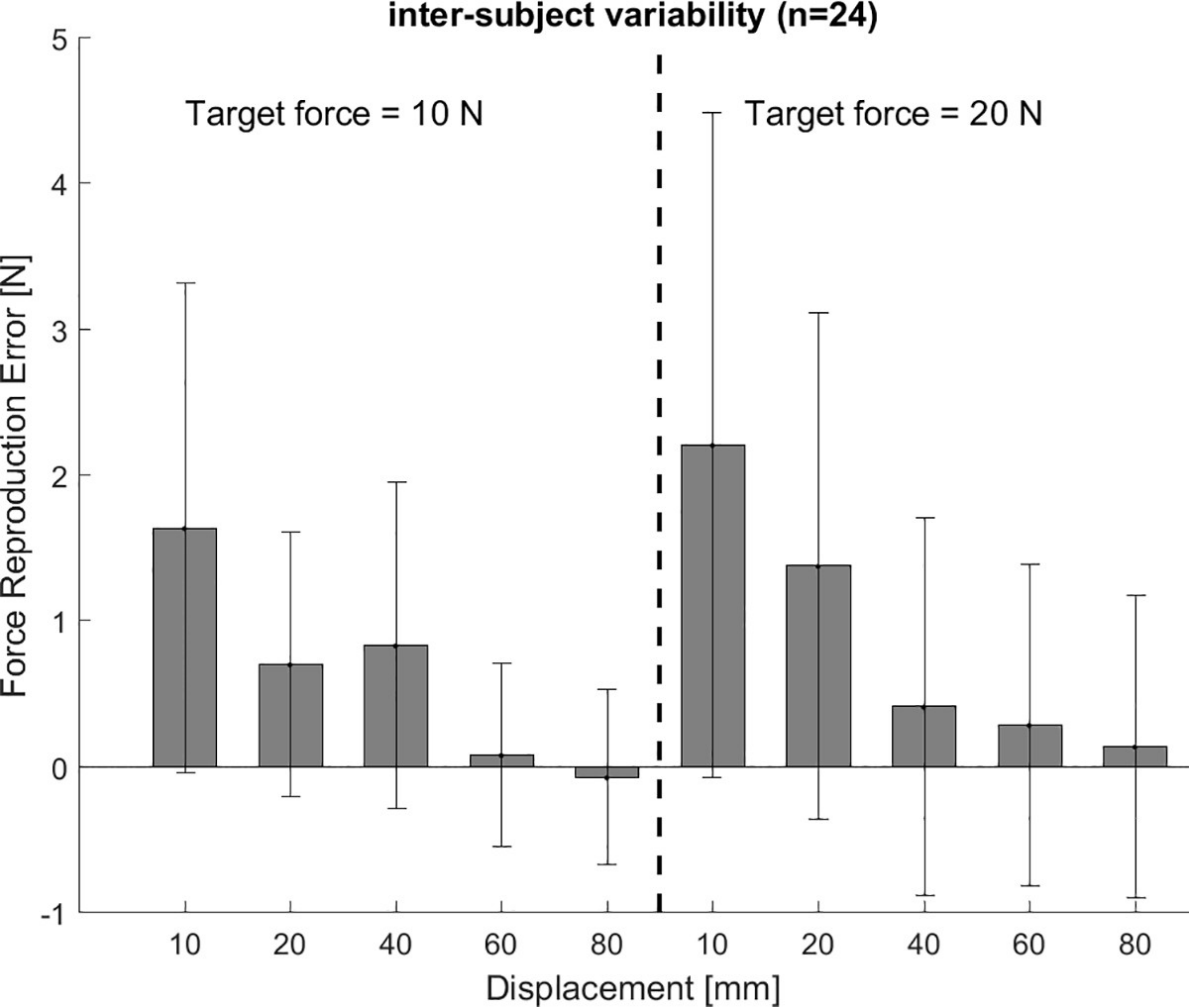
Force reproduction error ‘cable excursion’ trials. The force reproduction error decreases with increasing cable excursion for the ‘cable excursion’ trials for both target forces of 10 and 20 N. The force reproduction error does not differ between force levels. The zero line indicates when the target force is met. A negative force reproduction error indicates a lower reproduced force than target force. The bars indicate the group’s average and the whiskers the standard deviation.

**Fig 9:**
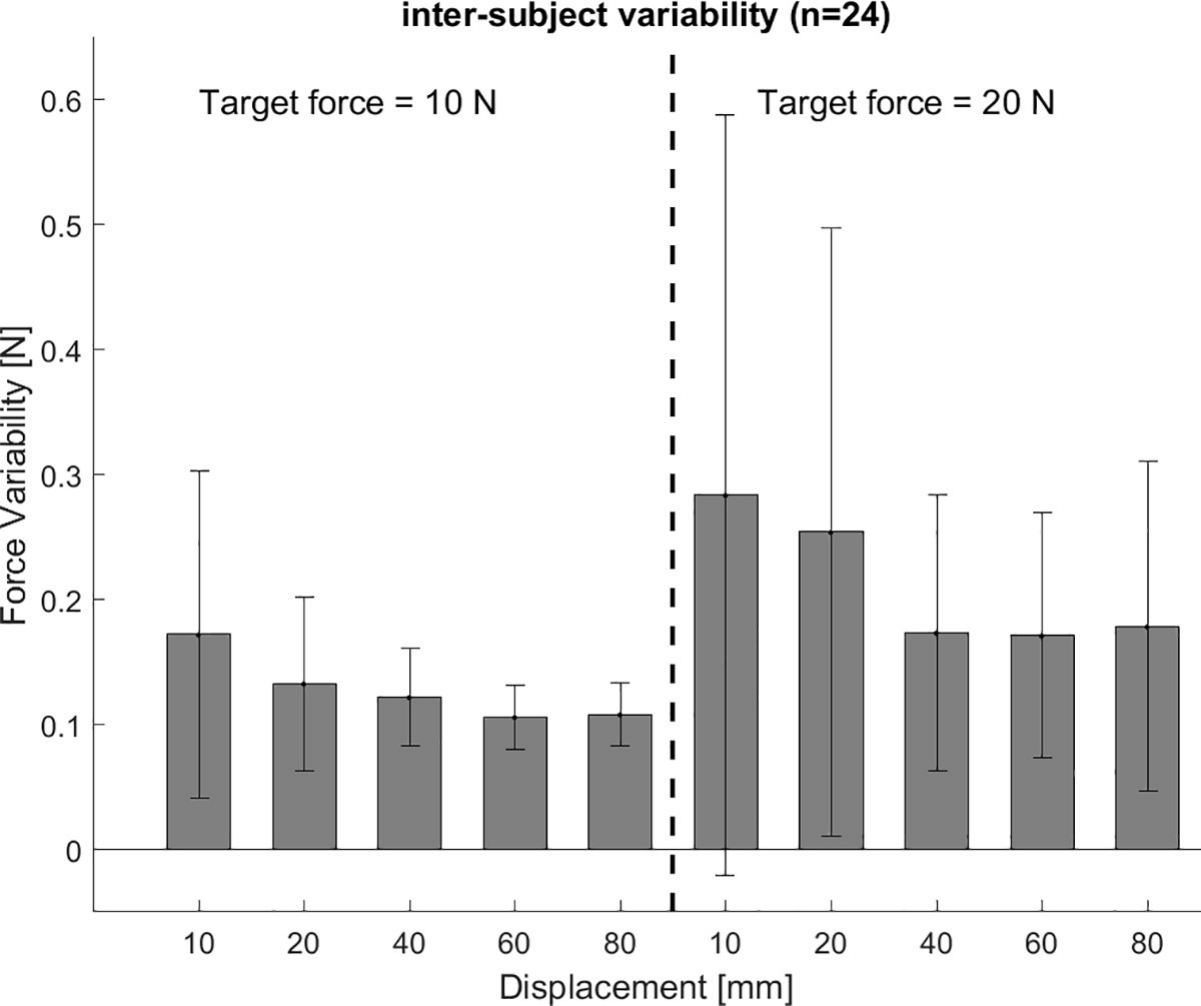
Force variability ‘cable excursion’ trials. The force variability remains constant with increasing cable excursion for ‘cable excursion’ trials. The force variability is lower for 10 N target force than for 20 N. The bars indicate the group’s average and the whiskers the standard deviation.

In the ‘cable excursion’ trials the force reproduction error was decreasing with increasing cable excursions for both target forces 10 and 20 N (F(4,20) = 8.865, p<0.001) (Fig 8), whereas no difference in force variability was found for increasing cable excursions at both target forces(F(4,20) = 1.878, p = 0.154) (Fig 9). No difference in force reproduction error between 10 and 20 N target forces was found for the ‘cable excursion’ trials (Fig 8). The force variability increases for increasing target forces (F(1,23) = 9.576, p = 0.05) for the ‘cable excursion’ trials (Fig 9). The target force was overestimated for all conditions, except the 10 N – 80 mm condition. As a result, we found a crossover point from over- to underestimation for a target force of 10 N between 60 and 80 mm cable excursion, whereas we did not find a crossover point for a target force of 20 N.

The pre and post maximum force measurements did not differ (T(24) = -0.50876, p = 0.61557), pre: 260.9±122.3 N; post: 267.0±119.5 N.

### Subjective data

The NASA-TLX questionnaires did not show any differences between the tested conditions for the measured indexes (mental, physical and temporal demand, overall performance, frustration level and effort).

The Body Maps showed that maximal force tasks provoked discomfort and pain in many of the subjects. Regardless of whether these maximum force measurements occurred at the start of the experiment or at the end, four of the twenty-four subjects reported pain (red) in neck, upper back, shoulder or axilla, eight reported irritation (orange) and fifteen reported touchiness (green). Interestingly, the third Body-Map showed that multiple subjects also experienced pain, irritation and touchiness at the low forces in the ‘cable excursion’ trials, suggesting that long-term exposure of low forces also results in discomfort.

An email questionnaire on the following day revealed that fourteen subjects had no post-experimental pain the next day(s), eight reported that they had aching arms, shoulder or necks varying from light to heavy, and two did not reply.

## Discussion

Contrary to our expectations, we did not find a difference in force reproduction error for measured target forces between 10 and 40 N during the ‘no cable excursion’ trials, whereas the force variability increased with increasing target forces, as hypothesized. The ‘cable excursion’ trials showed, as hypothesized, a decreasing force reproduction error with increasing cable excursions, for both target forces 10 and 20 N, whereas we did not find any difference in force variability at increasing excursions. The target forces were overestimated for all conditions, except for the 10 N -80 mm condition.

The *‘no cable excursion’ trials* were designed to simulate grasping a rigid object with a voluntary closing body-powered prosthesis. Of course, the compliance of the prosthetic system is always present. This compliance is similar between the prosthesis and the simulator used in this study as the harness, the Bowden-cable, and the couplings are typically the same. When grasping a rigid object with a voluntary closing body-powered prosthesis, the pinch force increases proportionally with the cable force. This occurs without a change in cable excursion and thus a change in prehensor opening. The increasing force variability for increasing target forces indicates a higher deviation of cable forces for higher forces. As a result, the deviation of pinch forces on an object increases with increasing required pinch forces. The controllability of pinch forces therefore decreases for increasing force levels. With current voluntary closing prostheses this implies less control on for example heavy objects where higher pinch forces are required. When a pinch force exerted on an object is too small the object might slip off the prehensor and fall, when the pinch force is too high the object might break. Both results in unsuccessful object manipulation, which discourages the user to manipulate objects with his prosthesis. We expected a crossover point from over- to underestimation around 10 to 20 N, as in preliminary experiments obtained for prosthesis users [13], but surprisingly the overestimation did not decrease with increasing force levels. This might be explained by a shorter force reproduction duration and less repetitions per force level compared to the experimental procedure in the preliminary experiments [13]. The overestimation of target forces indicates that the exerted pinch force on a rigid object would be higher than intended by the body-powered prosthesis user at low cable operation forces. However, for the tested force levels the offset of estimated and produced force on an object is expected to remain constant, based on the unchanged force reproduction error. The relationship between cable operation and pinch force can be described by F_pinch_ = k*F_cable_ for voluntary closing body-powered prostheses. Since the proportionality constant k is smaller than 1 for current voluntary closing prostheses [3], the effect of the overestimated cable force is smaller for the pinch force. For example: the proportionality constant k for the TRS hook is 2/3 [3]. A deviation of ±3 N in cable force results in a deviation of ±2 N in pinch force. Overall the force reproduction error has a larger impact than the force variability for the measured forces up to 40 N, ±3 N versus ±1.5 N. In other words, for the manipulation of light objects the difference between estimated and produced pinch force has a larger impact than the ability to hold a pinch force at a constant level.

The *‘cable excursion’ trials* were designed to simulate approaching a desired pinch force to hold an object with a voluntary closing body-powered prosthesis. Building up the cable force and increasing the cable excursion closes the voluntary closing prehensor. When the voluntary closing prehensor is fully closed or touches an object a pinch force is created. From the experimental results of the ‘cable excursion’ trials we learned that increasing cable excursions may help to estimate and approach the desired pinch force more accurately. Increasing cable excursions do not affect the deviation of produced pinch forces. This implies better control of pinch forces when the voluntary closing prosthesis requires a long stroke to close the device. Or, since smaller objects require a longer closing stroke than larger objects, the pinch force on small objects can be controlled better than on large objects with a voluntary closing prosthesis. Contrary, in a voluntary opening prosthesis the pinch force on large objects can be controlled better than on small objects, since it requires a longer stroke to fully open the device to be able to grasp the large object. This can be beneficial when manipulating fragile objects with a voluntary opening prosthesis. Here the pinch force created by the spring needs to be counterbalanced by the applied cable force in order to not break the fragile object.

However, although cable excursions of 80 mm show the lowest force reproduction error it is questionable whether large cable excursion is feasible for practical use. Utilizing this amount of cable excursion during grasping tasks implies that the prosthesis has to be held far away from the body, which makes object manipulation impractical, especially during feeding tasks. Whether trans-humeral patients might be able to apply these long strokes is worth further investigation. Current voluntary closing and voluntary opening body-powered prostheses demand cable excursions of up to 53 mm to fully close or open the prehensor respectively [3,4], and therefore we consider cable excursions of 53 mm as clinically approved. Practicality of cable excursions higher than 53 mm need to be examined in daily activities before recommending it for body-powered prosthesis design. Cable routing across the lateral epicondyle enables users to operate a voluntary closing prosthesis with elbow flexion and might increase the functional cable excursion. Additionally, an alternative harness system like the Anchor System [24,25] might facilitate larger cable excursions in clinical practice.

Interestingly, differences in the measured forces at the back and at the forearm of participants typically ranged between 2 to 3 N, but incidentally even up to 9 N. Such differences occur due to friction losses of the Bowden cable. However, irrespective of the magnitude of friction losses we found significant differences in the force reproduction error between conditions for the ‘cable excursion’ trials. Hence, the magnitude of friction should not have influenced the outcome of these experiments.

Note that in the present study we provided visual feedback of the force measured at *the forearm*; whereas subjects received proprioceptive feedback from back muscles. We chose to provide feedback of the force measured at the forearm of the subject to the screen, since this cable force is directly related to the created pinch force of the voluntary closing body-powered prosthesis [3]. After all, the user gets visual information of the created pinch forces when manipulating deformable objects with his prosthesis.

The experiments imitate intensive prosthesis use. All subjects could complete the full experiment, which suggests that the tested range of 10 to 40 N cable operation force is feasible for daily prosthesis operation. Interestingly, although pre and post experimental maximum force measurements did not show differences, eight subjects reported post experimental pain the next day(s). Furthermore, not only the magnitude of applied forces (maximum force measurements), but also the duration of the experiment seemed to provoke discomfort and pain as indicated by the results of the Body-Maps. Unfortunately, the subjective data of the Body-Maps does not include the severity of the pain, which makes interpretation of this subjective data difficult.

The perceived workload reported in the NASA TLX questionnaires did not differ between conditions. This implies that subjects do not show any preference for one of the tested force or force-excursion combinations.

### Study limitations

We only tested for force levels between 10 and 40 N. In preliminary experiments we observed inferior control and perception of cable forces lower than 10 N. The observed friction losses in the Bowden cable probably also complicate control and perception of forces below 10 N proportionally more than for higher force levels. Force levels higher than 40 N would probably lead to fatigue during long-term operation. Of course in clinical practice the individual fatigue force level should be considered. Furthermore, cable excursions are only investigated for two low force levels of 10 and 20 N. Between these two force levels we expected the crossover point from over- to underestimation based on preliminary experiments [13], but we found over-estimation for all tested force levels.

A second limitation in our study was the rather abstract task, focused on obtaining results that could generalize over different prehensor types. Therefore, we chose to simulate prosthesis behavior by utilizing springs of different stiffness and disabling cable excursion by a thread-rod. Of course, this experimental set-up is different than manipulating objects with a body-powered prosthesis, but gave us the opportunity to test different prehensor settings to make an informed choice of voluntary closing body-powered prosthesis design parameters. Also the duration and intensity of the experiment were considerable. Participants were requested to reproduce a force at one force level with many repetitions in short time. Since the pre and post maximum force measurements did not show a significant difference, we conclude that the data was not influenced by physical fatigue effects, which is in line with the answers given in the NASA-TLX questionnaire. Also the long duration of the experiments (±2 hours) might have influenced the participants’ performance due to mental fatigue, although the NASA-TLX questionnaire did not indicate mental fatigue.

Severity of pain might have better been evaluated by using for instance a pain scale from 1 to 10 (1 = no pain, 10 = worst pain you can imagine).

### Further research and implications

Users of upper limb prostheses have shown a preference for electric hands and body-powered hooks (n = 242) [26]. Compared to body-powered hooks, body-powered hands require a high activation force and have a high energy dissipation [3,4] and are probably therefore often rejected. The minimum realistic activation forces of body- powered hands do not allow them to be operated with cable forces as low as those measured in this study. Cosmetically, however, prosthetic hands seem to be more appealing than hooks. A possible solution might be found in introducing power assistance systems to body-powered hands. The results of this study could serve as input for new body-powered prosthetic hand designs. The operating force should be within the 10-40 N range, while maximizing the operating cable excursion. The implications for the mechanical advantage or proportionality constant need further study. Output requirements of such a system in terms of desired pinch forces for daily activities remain unknown.

## Conclusion

The aim of this study was to quantify the accuracy of cable force perception and control, when using a body-powered prosthesis with a low cable operation force range, by means of (isometric and dynamic) force reproduction experiments. For the experimental conditions studied, the following can be concluded:

- Contrary to our first hypotheses, force reproduction accuracy did not depend on the tested force levels (10 – 40 N): during the isometric force reproduction task, the target force was consistently overestimated, regardless of the tested force level.
- As hypothesized, force variability due to motor noise significantly increased with increasing force levels.
- As hypothesized, the presence of cable excursions contributes to higher force accuracy, as compared to isometric force reproduction.

When translating cable forces proportionally to pinch forces of a voluntary closing body-powered prosthesis the results imply a higher deviation of pinch forces at higher force levels due to motor noise. The estimation error of created pinch forces on rigid objects does not vary for the examined low force levels, but the created pinch force is constantly higher than intended. A long closing stroke for voluntary closing body-powered prosthesis accommodates the right estimation of pinch forces on objects. In conclusion, an operation force of 10-40 N is desired for future prosthesis design, since it offers fatigue-free operation with the best possible perception and control of the applied pinch forces. Increasing cable excursions up to 80 mm increase the accuracy of the estimated force.

## Supporting information

S1 Equation Force reproduction error block(i). (DOCX)

S2 Equation Force reproduction error. (DOCX)

S3 Equation Force variability block(i). (DOCX)

S4 Equation Force variability. (DOCX)
