# Supplementary material for: Perception and control of low cable operation forces in voluntary closing body-powered upper-limb prostheses
Source: PLoS One. 2019 Nov 22;14(11):e0225263. doi: 10.1371/journal.pone.0225263 (PMC6874327; doi:10.1371/journal.pone.0225263)
Supplement: Supplementary file 3 [file pone.0225263.s003.docx]

$$FV_{block_i} = std(F_{blind,block_i})$$
